# Automatic composition of Guzheng (Chinese Zither) music using long short-term memory network (LSTM) and reinforcement learning (RL)

**DOI:** 10.1038/s41598-022-19786-1

**Published:** 2022-09-22

**Authors:** Shuling Chen, Yong Zhong, Ruxu Du

**Affiliations:** grid.79703.3a 0000 0004 1764 3838 S. M. Wu School of Intelligent Engineering, South China University of Technology, No. 777 Xingye Road East, Panyu District, Guangzhou, 511442 China

**Keywords:** Computer science, Computational science, Information technology

## Abstract

In recent years, with the advance of Artificial Intelligence, automatic music composition has been demonstrated. However, there are many music genres and music instruments. For a same piece of music, different music instruments would produce different effects. Invented some 2500 years ago, Guzheng is one of the oldest music instruments in China and the world. It has distinct timbres and patterns that cannot be duplicated by other music instruments. Therefore, it is interesting to see whether AI can compose Guzheng music or alike. In this paper we present a method that can automatically compose and play Guzheng music. First, we collect a number of existing Guzheng music pieces and convert them into Music Instrument Digital Interface format. Second, we use these data to train a Long Short-Term Memory (LSTM) network and use the trained network to generate new Guzheng music pieces. Next, we use the Reinforcement Learning to optimize the LSTM network by adding special Guzheng playing techniques. Comparing to the existing AI methods, such as LSTM and Generative Adversary Network, our new method is more effective in capturing the characteristics of Guzheng music. According to the evaluations from skilled Guzheng players and general audiences, our Guzheng music is very close to the real Guzheng music. The presented method can also be used to automatically compose the music of other Chinese music instruments.

## Introduction

Music is a universal art enjoyed by all the people around the world. It is well known that music has different genres and can be played using different music instruments. Music composition has always been considered as creative art that only talented musicians can master. In recent years, the technology of Artificial Intelligence (AI) has been greatly advanced. There have been a number of successful attempts to use AI for music composition. However, the success is rather limited as only a few kinds of music, such as piano music and guitar music, can be generated. Particularly, there has been no reported success in composing traditional Chinese music.

Among various traditional Chinese musical instruments, Guzheng (Chinese Zither) (古筝) is perhaps ranked at the top in popularity. Invented some 2500 years ago^1^, through years of inheritance and innovation, development and improvement, it has its unique music language, playing style, as well as special identity unlike any others. In fact, just hearing Guzheng music, many people around the world would think of China.

**Figure 1:**
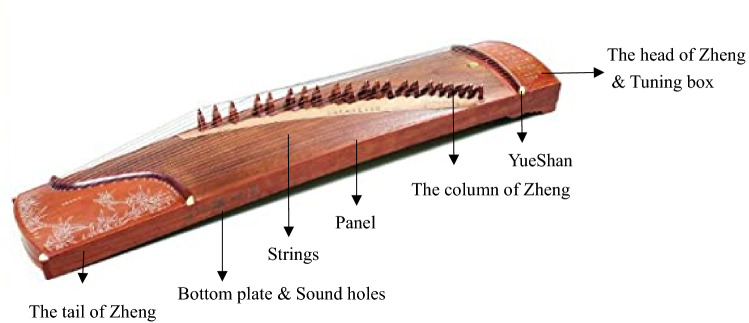
Guzheng, a traditional Chinese music instrument (Free of copyright).

Figure 1 shows the picture of a classical Guzheng. Its body is a hollow box made of sycamore wood. Its strings are placed on the top face while the sound holes are at the bottom (not shown in the figure). From the structure point of view, Guzheng consists of 5 parts: (1) the head of the Guzheng and tuning box, (2) Yue Shan (岳山) on which the strings are mounted, (3) the column of Guzheng that acted as the bridge for the strings, (4) the panel, on which the strings are crossed, and (5) the tail of Guzheng. According to the historical records, the original Guzheng had only 5 strings. In Three-Kingdom period (220–280), it had 12 strings. In Tang Dynasty (618–907), it added to 13 strings, and in Ming Dynasty (1368–1644) and Qing Dynasty (1636–1912) it had 16 strings. In the modern era, it was sublimated to modern musical instrument making method and gradually increased to 18 strings, 21 strings and even 25 strings. Presently, the design of Guzheng is standardized with 21 strings and a fixed length of 1.63 m long. Its tone is in pentatonic scale with a range of three octaves. The mode is mostly G or D. Also, players need to wear tortoise’s bill armor pieces (i.e., false fingernail) to play.

There are a number of special skills for playing Guzheng. The right hand uses the thumb, index finger, middle finger and ring finger to pluck the strings. The fingering methods include Gou (勾), Tuo (托), Pi (劈) and etc. The left hand presses the strings and the fingering techniques include Press (按), Slide (滑), Chatter (揉) and so on. In recent years, new Guzheng playing techniques are also added such as playing the left and right hand alternatively and playing multi-part together. It creates very rich and vibrant music^2–4^.

There are at least 200,000 people are playing Guzheng with different skill levels. In 2019, there were over 17,000 people participating the national exam, making it the most popular traditional Chinese music instrument. Indeed, many people view Guzheng as important inheritance and have special cultural value. In recent years, although the effort of Guzheng music composition also increases, few music pieces could not reach the height of the traditional Guzheng music pieces. Therefore, it is wondrous if we can use AI to compose good Guzheng music.

AI based music composition, also referred to as computer music generation, refers to generate a specific sequence of music notes with minimum human intervention. Based on literature survey, the first attempt of computer music generation can be dated back to 1950s^5^. The early pioneers proposed to use Markov model to generate music, but the experiments showed that only fragment musical notes were generated. Later research found that the Markov chain model offers only a stitching process and cannot be called music composition. Consequently, Hidden Markov Model (HMM) were proposed which was able to generate polyphony music for the first time^6^. However, HMM has some limitations, such as its state evolution is linear, it is difficult to learn long-term dependence, and it is incapable of dealing with large data sets.

Neural network is another commonly used method for computer music generation^7^. For example, in^8^, Schuster and Paliwal used bidirectional Recurrent Neural Network (RNN) to generate computer music. However, because of the gradient disappearance of RNN, it is difficult to learn the long-term correlation within a music piece, resulting in fragmented note cycles. In^9^, Hang and Urtasun used Long Short-Term Memory network (LSTM) to generate more complex music. LSTM can memorize long-term information and avoid the aforementioned gradient disappearance problem. Consequently, it makes the generated music pieces more coherent. In^10^, a new LSTM with Gated Recurrent Unit (GRU) was used obtaining improved results. Subsequently, LSTM is widely used in computer music generation^11–13^, so we pick LSTM to generate Guzheng melody. In^14^, in order to solve the problem of melodic level of music generated by LSTM, an algorithm called Music VAE (Variational Autoencoder) is proposed. In^15^, VAE is used to improve the quality of audio synthesis. The emerging of Generative Adversarial Networks (GAN) algorithm^16^ provides a new method for AI based music composition. GAN consists of two networks: the generating network G (Generator) and the discriminant network D (Discriminator), the training achieves a Nash equilibrium, making G capable of generating data with the same distribution as that of the training data and D capable of detecting false data, which is ideal for music composing. The used of GAN for music composition is reported in^17,18^, though, it seems the generated music pieces are still full of random notes.

Reinforcement Learning (RL) is another common method for AI music generation. In^19^, Kotecha used a two-layer bi-axial LSTM with deep reinforcement learning (DQN) aimed for better global coherence. Though, the generated music pieces are rather short and assessed by only one music professional with vague comments. In^20^ Jiang and et al. used RL for interactive music generation. Again, the generated music pieces are short and lack of fluency. In^21^ Karbasi and et al. used RL to model musical rhythms, however, the rhythms are all quite simple, and the agent seems to be blind to the dynamical behavior of the environment. In^22^ Jaques et al. developed a new RL method for fine-tuning the generation models with KL (Kullback Leibler)-control. They used music generation as an application example. Unfortunately, the generated music pieces contain many repeated notes.

In short, there are a number of common problems for automatic music composition, First, it is clear that the brute searching and matching of the existing AI methods could only result in poor imitation. Second, there is a lack of subjective and objective evaluation method for generated music. Given the fact that most of the generated music pieces are rather short and lack of rhythm and fluency, there is still a long way to go.

Moreover, no one has tried to use AI to generate Guzheng music. This may be attributed to several challenges. Firstly, it is known that the modern digital music format is MIDI. Currently, the MIDI library contains the timbres of many music instruments, such as piano, violin, guitar etc., but not Guzheng. Consequently, it becomes difficult to generate Guzheng music. One may of course use MP3 format, though, it requires much more training samples and computation loads. Yet, it cannot guarantee the success. Secondly, Guzheng has many unique playing skills, which cannot be readily expressed in MIDI format. Third, simply apply the AI methods for Guzheng music generation may not work. This is because Guzheng music has distinct characteristics created by is its unique playing techniques. These playing techniques cannot be further decomposed and hence, mock up. Also, there are few people who know both AI and Guzheng.

In this paper, we present a new method for composing Guzheng music using LSTM and Reinforcement Learning (RL). It consists of three steps: preprocessing, training and music generation, as well as post processing. Accordingly, the rest of the paper is organized as follows.  “Pre-processing” section describes the pre-processing steps including a brief review of music, the conversion of the Guzheng music to MIDI format, as well as the extraction of chorus. “Training using LSTM and RL” section discusses the core of our method: training using LSTM and Reinforcement Learning (RL). “Post-processing and experiment results” section  shows the post-processing step as well as the experiment results. Finally, “Conclusions and future work” section contains conclusions and future work.

## Pre-processing

### A brief review on the elements of music

There are several different views on the elements of music. From the acoustic point of view, music is made of sounds which can be characterized by its time and frequency characteristics. From the music point of view, though, the definitions vary. The commonly accepted definition is that the basic elements of music include pitch, duration, loudness, timbre, sonic texture and spatial location. Though, the most important elements of music are timbre and melody.

**Figure 2:**
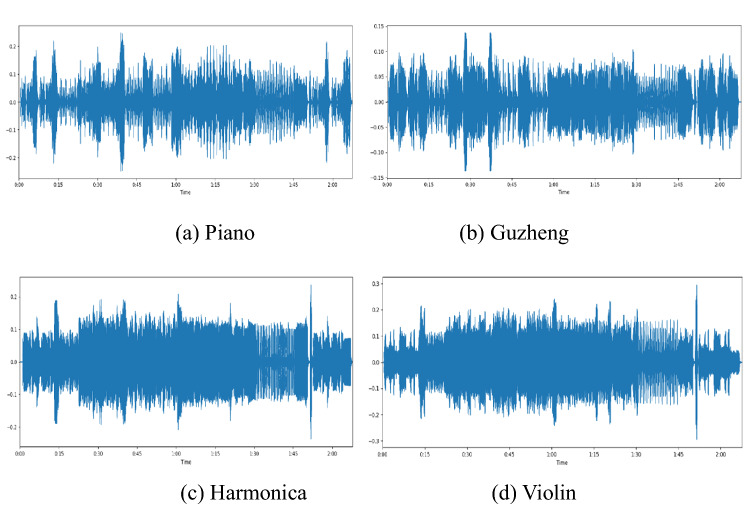
The sound waves of different music instruments when playing a same piece of song with the same volume.

Timbre is the perceived sound quality of a musical note, a sound or a tone. It distinguishes a sound from the others. Timbre can be characterized by the following attributes: the tone (the arrangement of pitch and/or chord), the spectral envelop, the time envelop, the changes of the frequency and spectral envelop, as well as the onset of the sound^23^. Guzheng, like the other music instruments, has its unique timbre. Figure 2 shows the sound wave from Piano, Guzheng, Harmonica, and Violin when playing a same piece of song with the same volume. As expected, Guzheng has unique timbre different to all others.

Melody, also called tune, is a timely succession of music tones. It is a combination of pitch and rhythm. Melody often consists of one or more musical phrases and are usually repeated within the music piece. Guzheng music belongs to traditional Chinese music and has its own melody styles and patterns. In particular, its emphases on artistic conception and cavity rhyme. As shown i ^24^, traditional Chinese music uses five tones: C, D, E, G, and H. Additionally, it usually uses single instrument, single rhythm. Though, modern Chinese music is more diversify and uses the standard seven tones. However, its artistic conception and cavity rhyme are difficult to describe.

In order to generate Guzheng music, it is necessary to sort out the timbre of the Guzheng as well as the melodies of the Guzheng music first.

### Guzheng music data pre-processing

Music data can be represented in two kinds of format: the audio format (MP3 or AVI) and the MIDI format. In this paper, we use the MIDI format as it is concise and easy to edit. MIDI files contain musical notes, timing, and playing definitions of up to 16 channels. For each channel, it includes channel number, length, volume, strength, etc. Because a MIDI file is a series of instructions, it requires little disk space. More importantly, one can easily add, delete or edit the attributes of a note.

**Figure 3:**
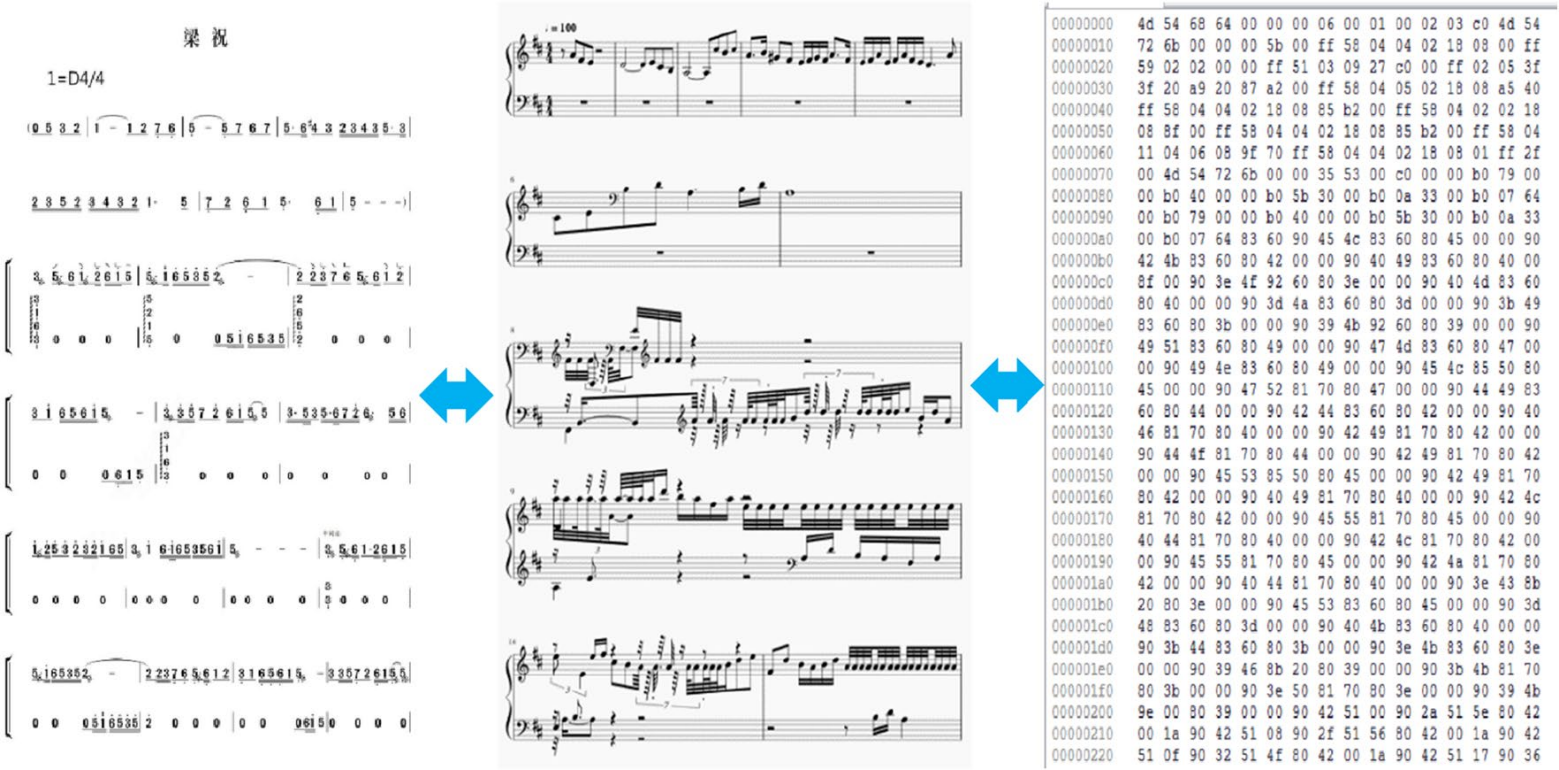
Music format conversion.

We pick Guzheng music pieces from the popular Guzheng repertoires in the Guzheng training textbooks. These music pieces are in the form of numbered notation. Next, we convert these music pieces to form of staff notation. Then, we use a freeware MuseScore to convert them to MIDI format. This process is illustrated in Fig. 3.

A total of 31 pieces of Guzheng music are used for training. These music pieces range from 30 s to 4 min in length. It shall be pointed out that MIDI has the play definitions of many music instruments, such as piano, violin, guitar and etc., but not Guzheng. From the mechanics point of view, Guzheng is similar to Guitar, which generate sounds with one hand pressing the strings while the other hand toggling the strings. However, Guzheng has 21 strings and can play register from D2–D6 (International phonetic alphabet) while Guitar can only play register from E2–C6. Therefore, we use piano to animate Guzheng, as it can play 88 notes, for music generation. The generated music will be converted to Guzheng music by converting to the Guzheng timbre in post-processing, which will be shown in Sect. 4.

### Extraction of the melodies

As pointed out in earlier, music is characterized by its melody. Thus, we extract the melodies of the training samples use them to enhance the training. In general, melody may appear in two ways: (1) For simple and short music, its melody is the chorus which replicates itself with minor variations. (2) For complicated and long music, its melody is characterized by its tensed motion and cadence.

For capturing the melodies, we use the similarity matrix. In the MIDI format, the music is scanned at a rate of 10 frames per second, or one frame every 0.1 s. Each frame is represented by a 128-dimensional vector giving 128 pitches in total. The value in each dimension represents the strength of the note in the corresponding pitch. Thus, each frame can be described by a vector, $v$ = [$x_{1}$, $x_{2}$ …, $x_{128}$]. Moreover, a piece of music can be described by a matrix *X* = [$v_{1}$, $v_{2}$, …, $v_{N}$]^T^ where, *N* number of frames. Accordingly, the similarity matrix is a (*N*-1) × (*N*-1) matrix, whose element is as follows:

1 $$ S_{i.j} = \sqrt {\left( {v_{i} - v_{i + 1} } \right)^{2} + \left( {v_{j} - v_{j + 1} } \right)^{2} } ) $$

where *i, j* = 1, 2, …, *N − *1.

**Figure 4:**
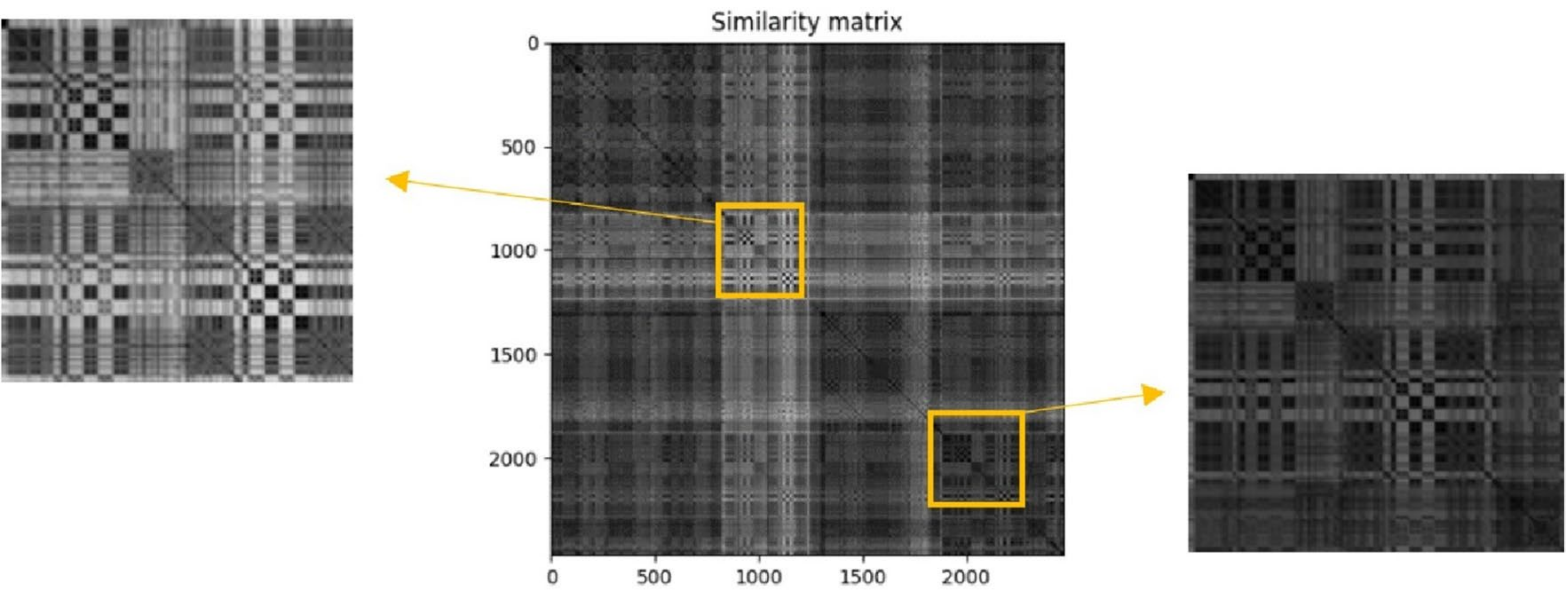
The similarity matrix of a sample music.

Figure 4 shows the similarity matrix of a piece of music. In the figure, different levels of brightness represent different levels of similarly. It is seen that the ones marked by yellow boxes have a similar pattern and hence, are correspondent to the melody.

The similarity matrix works fine for finding repeats with minor variations. For repeats with relatively large variations, we can use the similar index defined below:

2 $$ {\text{S}} = 1 - \frac{{\left\| {X_{i}  - X_{{i + 1}} } \right\|}}{{\surd 12}}  $$

where 12 corresponds to the number of basic notes, $X_{i}$ and $X_{i + 1}$ are the matrices corresponding to the two segments in the music piece. If the value of S is high, then the two segments are similar.

**Figure 5:**
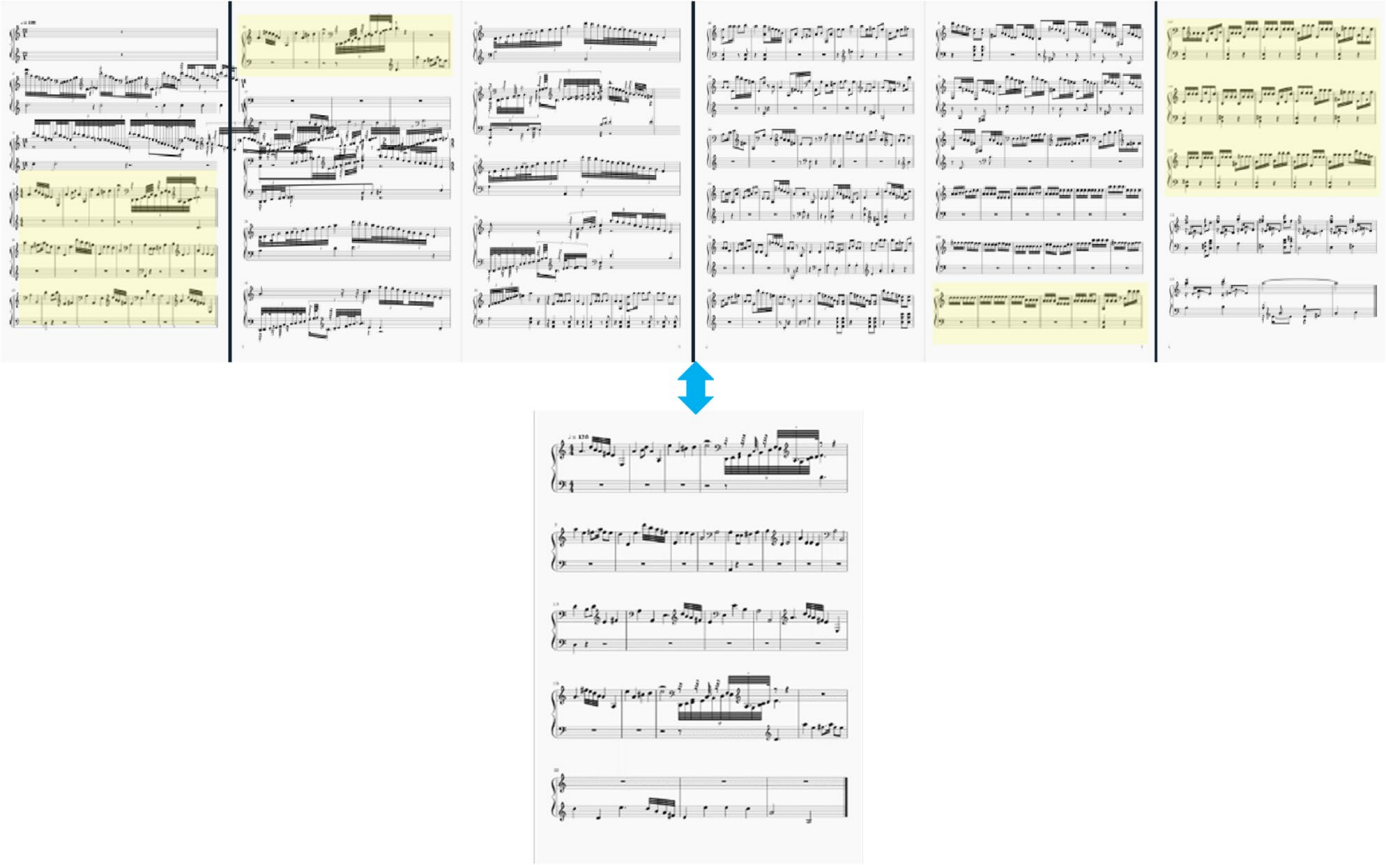
An example of melody extraction, the melody is marked by yellow.

As an example, Fig. 5 illustrates the extraction of a melody.

There are Guzheng music pieces that do not have repetitions, such as “Gao shan liu shui (高山流水)” and “Yu zhou chang wan (渔舟唱晚)”. In this case, we will select the segment with high intense and cadence.

## Training using LSTM and RL

### A brief introduction of LSTM network

**Figure 6:**
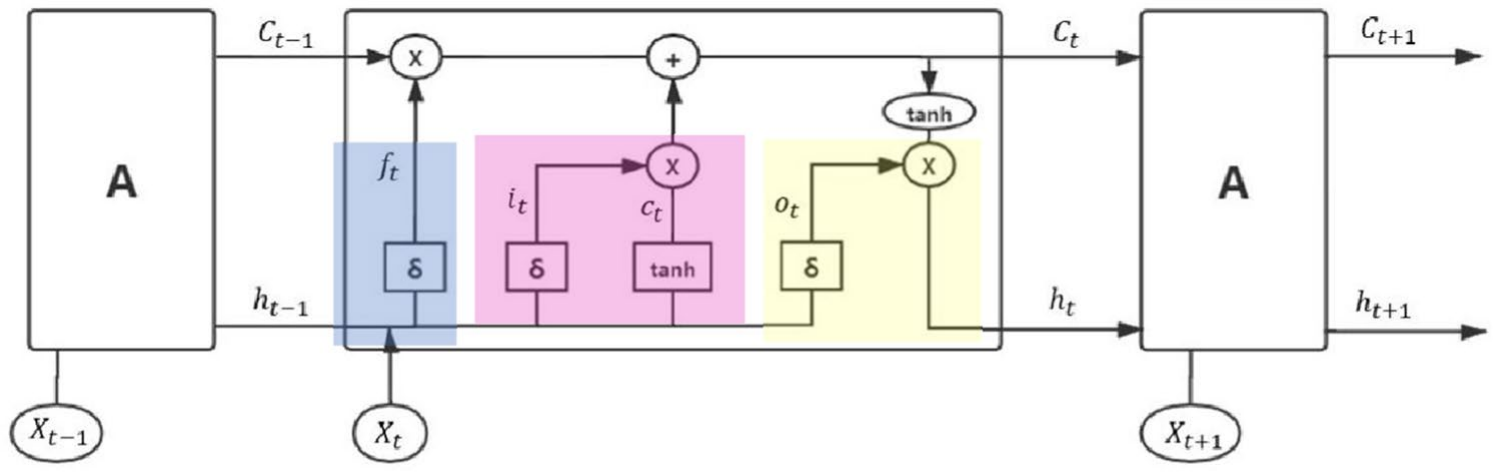
A LSTM unit.

In this paper, we use LSTM network for Guzheng music generation. Figure 6 shows a LSTM unit. It has three inputs: the data input (note vectors), $$X_{t}$$, as well as the outputs from the previous unit, $$h_{t - 1} $$ and $$ C_{t - 1}$$. Here, $$h_{t - 1}$$ is the output while $$ C_{t - 1}$$ represents how well the output shall be retained. As shown in the figure, the LSTM unit is made of three gates: the forgetting gate (in blue), the input gate (in pink) and the output gate (in yellow).

The forgetting gate is used to control how much information shall be “forgot” from the previous unit and is computed as follows:

3 $$ f_{t} = {\updelta }\left( {W_{f} * \left[ {h_{t - 1} ,X_{t} } \right] + b_{f} } \right) $$

where $$h_{t - 1}$$ is the output of the previous unit, $$X_{t}$$ is the input, $$W_{f}$$ is the weight, $$b_{f}$$ is the bias, and δ is the Sigmoid function. $$f_{t}$$ times $$ C_{t - 1}$$ determines how much information from the previous unit will retain.

The input gate is used to determine the effect of the inputs. It contains two parts:

4 $$ \begin{gathered} i_{t} = {\updelta }\left( {W_{i} {*}\left[ {h_{t - 1} ,X_{t} } \right] + b_{i} } \right) \hfill \\ c_{t} = {\text{tanh}}\left( {W_{c} {*}\left[ {h_{t - 1} { },X_{t} } \right] + b_{c} } \right) \hfill \\ \end{gathered} $$

where $W_{f}$ and $W_{i}$ are the weights, $b_{i}$ and $b_{c}$ are the biases.

The output gate is used to determine the information output. Just like the previous unit, it gives two outputs: $h_{t}$ is the output and $C_{t}$ represents how well $h_{t}$ shall be retain. The output $h_{t}$ is computed as follows:

5 $$ o_{t} = {\updelta }\left( {W_{o} {*}\left[ {h_{t - 1} { },X_{t} } \right] + b_{o} } \right) $$

6 $$h_{t}  = o_{t} *{\text{tanh}}\left( {C_{t} } \right) $$

where $W_{0}$ is the weight and $b_{o}$ is the bias. The control $C_{t}$ is computed as follows:

7 $$ C_{t} = f_{t} {*}C_{t - 1} + i_{t} {*}c_{t} $$

It is interesting to note that when $$f_{t}$$ → 1 and $$i_{t}$$ → 0, $$C_{t}$$ ≈ $$ C_{t - 1}$$, which retains all the information from the previous unit. On the other hand, when $$f_{t}$$ → 0 and $$i_{t}$$ → 1, $$C_{t}$$ ≈ $$c_{t}$$, which retains no information from the previous unit.

**Figure 7:**
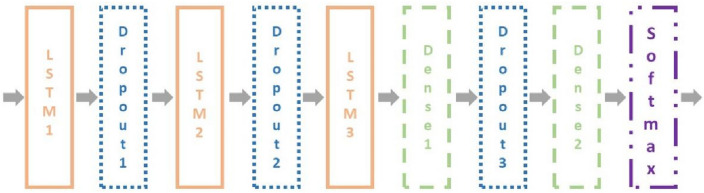
Model demonstration.

In this paper we use 3 LSTM, 2 Dropouts, 2 Denses and 1 Softmax as shown in Fig. 7. In the setting, the LSTM is described by three parameters: the sequence length, the characteristic number of the hidden layer, and the number of weights in the network. The Dropout is used to reduce overfitting by hiding 30% of the neurons. In the setting, it is described by two parameters: the sequence length, the characteristic number of the hidden layer. In the setting, it can be described by three parameters just like LSTM. The Softmax function is used to generate music notes. Given data sets, {$h_{j,t}$, *j* = 1, 2, …, *M*, *t* = 1, 2, …, *N*}, it first computes the probability:

8 $$ P\left( {h_{j,t} } \right) = \frac{{e^{{h_{j,t} }} }}{{\mathop \sum \nolimits_{j = 1}^{M} e^{{h_{j,t} }} }},j = 1,{ }2,{ } \ldots ,{ }M,{ }t = 1,{ }2,{ } \ldots ,{ }N $$

Then, the ones with the highest probability is selected as the generated note:

9 $$ y_{t} = \arg \mathop {\max }\limits_{j} P\left( {h_{j,t} } \right),{ }t = 1,{ }2,{ } \ldots ,{ }N $$

The training of LSTM is based on the minimization of the cross-entropy function:

10 $$ {\text{L}}_{1} = - \frac{1}{M}\mathop \sum \limits_{j = 1}^{M} \left( {Y_{j} {\text{log}}\left( {Y_{j} } \right) + \left( {1 - Y_{j} } \right){\text{log}}\left( {1 - Y_{j} } \right)} \right) $$

where $$Y_{j} = \frac{1}{128}\mathop \sum \limits_{t = 1}^{128} \left( {X_{j,t} - Y_{j,t} } \right)^{2}$$.

During the training, we set the learning rate as ${\upalpha } = 0.4{*}10^{ - 3}$. Also, considering the lack of contextual information in the first 6 notes of each musical piece, these notes are not be used.

In the setting, it can be described by the sequence length and the characteristic number of the hidden layer. In summary, Table 1 shows the network parameters.

**Table 1:** The network parameters.

Name	Setting parameters
LSTM1	The sequence length = 100; The characteristic number of the hidden layer = 512; The number of weights in the network = 1,052,672;
Dropout1	The sequence length = 100; The characteristic number of the chidden layer = 512;
LSTM2	The sequence length = 100; The characteristic number of the hidden layer = 512; The number of weights in the network = 2,099,200;
Dropout2	The sequence length = 100; The characteristic number of the hidden layer = 512;
LSTM3	The sequence length = 100; The characteristic number of the hidden layer = 512; The number of weights in the network = 2,099,200;
Dense1	The sequence length = 100; The characteristic number of the hidden layer = 256; The number of weights in the network = 131,328
Dropout3	The sequence length = 100; The characteristic number of the hidden layer = 256;
Dense2	The sequence length = 100; The characteristic number of the hidden layer = 138; The number of weights in the network = 35,466;
Softmax	The sequence length = 100; The characteristic number of the hidden layer = 138;

### An introduction of Guzheng playing techniques

Guzheng is a 2000 year old music instrument. Throughout the history, many playing techniques were added and modified. Currently, there are many complicated playing techniques, each has its own characteristics. It is impossible to list all the playing techniques, as new ones are continuously being invented and old ones are being modified. We therefore focus on the typical playing techniques instead. In general, the playing techniques can be divided into two categories: the left-hand playing techniques and the right-hand playing techniques.

The left-hand playing techniques imply the use of left-hand on the left side of the bridge. It changes the tension of the string, mainly by pressing the string, with the middle finger, ring finger or index finger. Its basic task is to decorate the play of the right hand, so that the tone is richer. Table 2 shows three typical left-hand playing techniques with their playing methods, usual location in a music piece and a sample timbre.

**Table 2:**
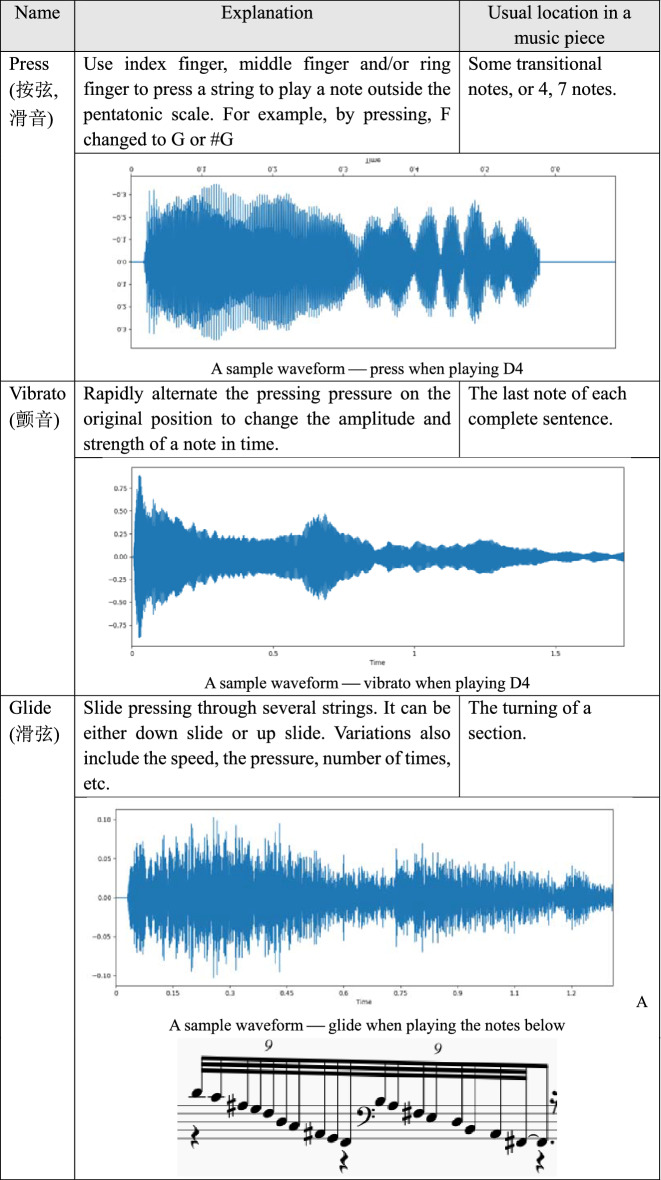
The left-hand playing techniques and their corresponding timbre example.

The right-hand playing techniques imply the use of right hand playing. Right hand playing is mainly to pluck the strings, and the position of the playing is mainly on the right side of the bridge. Its basic task is to play the tone. Depending on the music note, the player can use the index finger, the big finger, ring finger and/or middle finger to play. The key is to control the duration, loudness and sonic texture. Also, it is not unusual to play multi-tones at a same time. Table 3 presents four typical right-hand playing techniques with their playing methods, usual location in a music piece and a sample timbre.

**Table 3:**
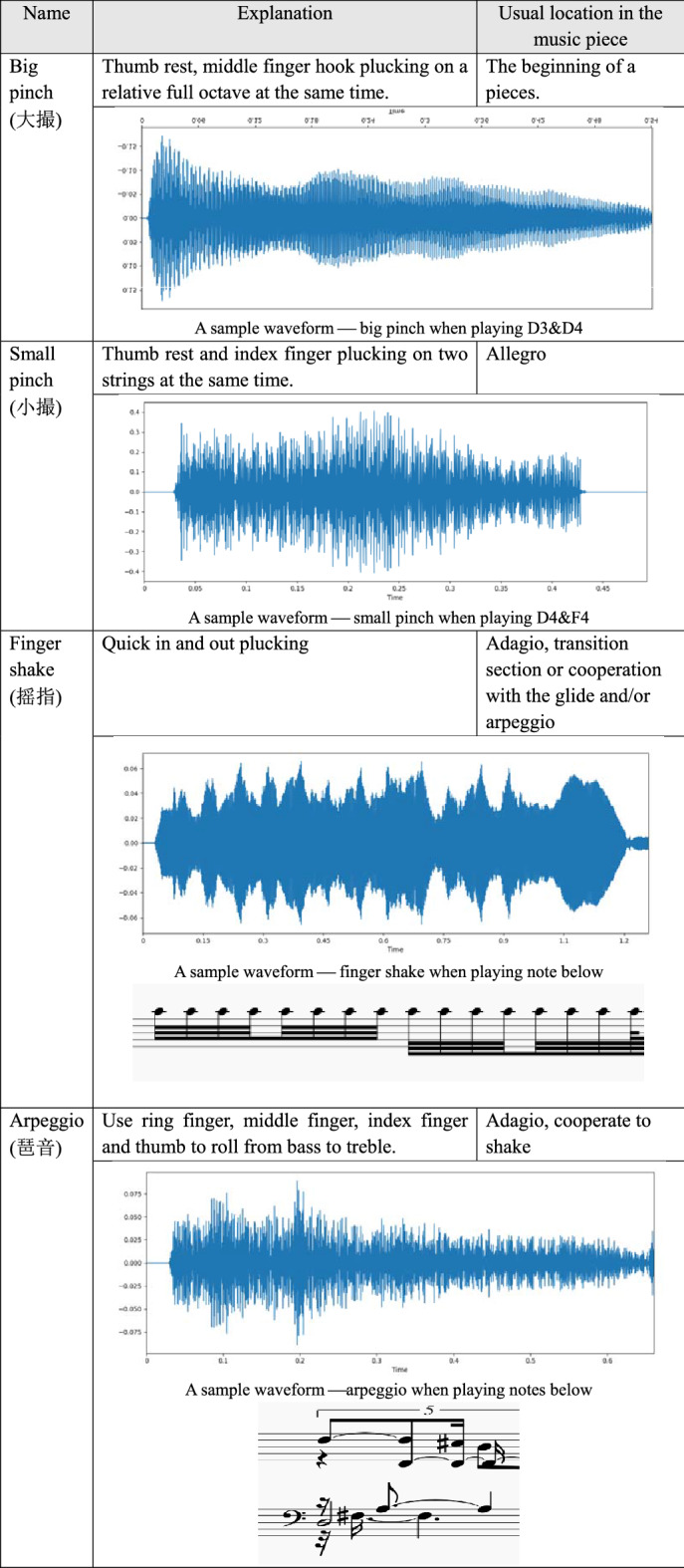
The right-hand playing skills and their corresponding timbre example.

### Adding the Guzheng playing techniques using RL

While LSTM network learns the dependence among notes from the dataset, it may not catch the characteristics of Guzheng music. Thus, we use Reinforcement Learning (RL) to add the Guzheng playing techniques to the music generated by the LSTM network.

**Figure 8:**
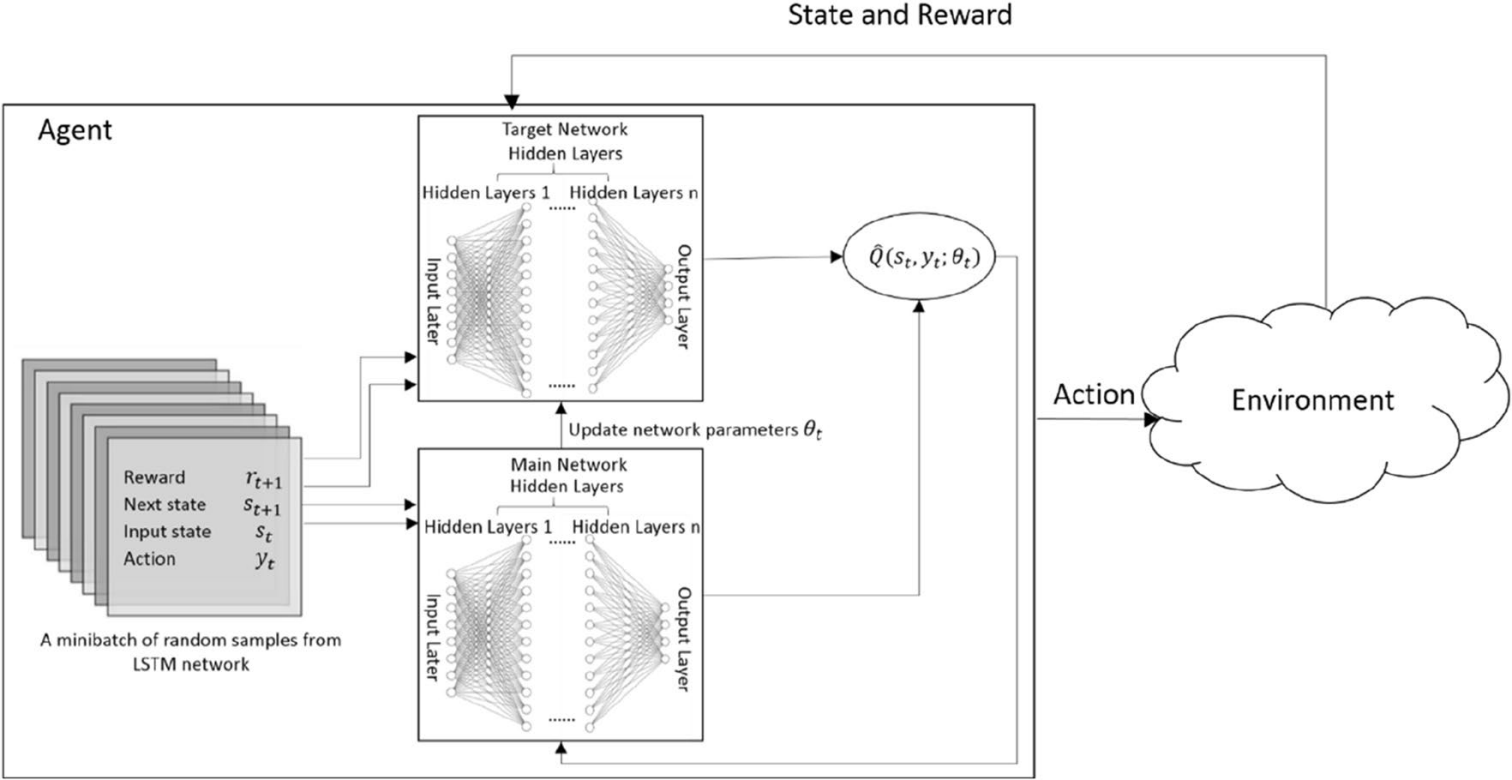
The RL process of agent-environment interaction in DQN.

The theory of RL has been well developed^25,26^. Figure 8 shows the agent-environment interaction in the RL process. The minibatch is a collection of random samples from LSTM network, the main network produces possible actions and the target network produces a stable target used to compute the loss of the selected action. At time *t*, following a policy, $\pi$, and the given the current state, $s_{t}$, the agent generates an action, $y_{t}$. According to the action, a reward, $r_{t + 1}$, and a new state, $s_{t + 1}$, will then be generated through the environment, which in turn will be input to the agent again in the next round. This process continues until satisfactory result is obtained.

We use Deep Q-learning Network (DQN) for RL. It is a model-free algorithm based on Q-learning and its goal is to select appropriate policies to optimize the sum of rewards at the end of each epoch. We use Deep Neural Network (DNN) for the main network, i.e.:

11 $$ y_{t} = \pi \left( {s_{t} } \right) = DNN\left( {s_{t} } \right) $$

The reward policy is designed to catch the characteristics of Guzheng music. It is made of a number of rules. Each rule has a positive reward and a negative punishment. The rule that has the strongest impact will be set to + 1 or − 1, and the remaining rules will be compared to the strongest rule to set the reward values.

As an example, let us consider Arpeggio (refer to Table 3). It is a technique usually consisting of seven notes. Many even-beat positions in Guzheng music end with arpeggio. Thus, assuming that at time *t*, the input state is $s_{t} = \left\{ {A_{t}^{1} ,A_{t}^{2} ,A_{t}^{3} ,A_{t}^{4} ,A_{t}^{5} ,A_{t}^{6} ,A_{t}^{7} } \right\}$, where $A_{{\text{t}}}^{1} ,A_{{\text{t}}}^{2} , \ldots ,A_{{\text{t}}}^{7}$ represents the seven notes incur at *t*, then, the reward policy is defined below:

12 $$ r_{t}^{1} \left( {s_{t} ,y_{t} ,t} \right) = \left\{ {\begin{array}{*{20}c} {0.2} & {if y_{t} \in \left\{ {A_{1}^{t} ,A_{2}^{t} , \ldots ,A_{7}^{t} } \right\} and{ }\left( {t = even beat} \right) { }} \\ { - 0.2} & {otherwise} \\ \end{array} } \right. $$

The other example is finger shake technique. We consider that when 4 same notes occur, it is the finger shake. However, if there are more than six same notes, it is considered that the notes generated are not vivid and smooth, giving people a bad experience. The reward policy, $r_{t}^{2}$, is to reduce the velocity by half to make it sounds more like finger shake:

13 $$ r_{t}^{2} \left( {s_{t} ,y_{t} ,t} \right) = \left\{ {\begin{array}{*{20}c} {0.3} & {if{ }y_{t} \in \left\{ {A_{{\text{t}}}^{1} = A_{t - 1}^{1} = A_{t - 2}^{1} = A_{t - 3}^{1} } \right\}} \\ {\begin{array}{*{20}c} 0 \\ { - 1} \\ \end{array} } & {\begin{array}{*{20}c} {if y_{t} \ne y_{t - 1} } \\ {if y_{t} \in \left\{ {A_{{\text{t}}}^{1} = A_{t - 1}^{1} = A_{t - 2}^{1} = A_{t - 3}^{1} = A_{t - 4}^{1} = A_{t - 5}^{1} } \right\}} \\ \end{array} } \\ \end{array} } \right. $$

As for big pinch and small pinch, they both contain two notes. Big pinch usually appears in downbeat while small pinch usually appears in upbeat. Thus, the reward policy is

14 $$ r_{t}^{3} \left( {s_{t} ,y_{t} ,t} \right) = \left\{ {\begin{array}{*{20}c} {0.7} & {if y_{t} \in \left\{ {A_{1}^{t} ,A_{2}^{t} } \right\} and \left( {t = odd beat} \right) } \\ { - 0.3} & {othewise} \\ \end{array} } \right. $$

In general, the total reward is:

15 $$ R_{t} = \mathop \sum \limits_{j = 1}^{n} r_{t}^{j} \left( {s_{t} ,y_{t} ,t} \right) $$

where *n* is the number of Guzheng characteristics. Assuming that the previous total return is:

16 $$ R_{total} = R_{t} + \gamma R_{t - 1} + \gamma^{2} R_{t - 2} + \cdots + \gamma^{n - t} R_{1} $$

where $\gamma \in \left[ {1,0} \right]$ is a discount value. Then, the average rate of return, $\overline{{R_{t} }}$, is:

17 $$ \overline{{R_{t} }} = \frac{1}{t}\mathop \sum \limits_{i = 1}^{t} \frac{{R_{t} }}{{R_{total} }} $$

Depending on the action, $y_{t}$, the value of the action is as follows:

18 $$ Q\left( {s_{t} ,y_{t} ;\theta_{t} } \right) = \mathop {\max }\limits_{{y_{t} }} \left( {s_{t} ,y_{t} } \right) $$

where${ }\theta_{t}$ is the weights of the DNN at time *t*.

Moreover, the target *Q*-value, $\hat{Q}\left( {s_{t} ,y_{t} ;\theta_{t} } \right)$, can be calculated by continuously updating the right-hand side of the formula below:

19 $$ \hat{Q}\left( {s_{t} ,y_{t} ;\theta_{t} } \right) \leftarrow \left( {1 - \alpha } \right)Q\left( {s_{t} ,y_{t} ;\theta_{t} } \right) + \alpha [R_{t} + \gamma \mathop {\max }\limits_{{y_{t} }} Q\left( {s_{t + 1} ,y_{t} ;\theta_{t} } \right)] $$

where ${ }\alpha \in \left[ {1,0} \right]$ and $\gamma \in \left[ {1,0} \right]$ are the parameters for controlling the effects of the previous and current actions, and $s_{t + 1}$ is the new state. In this study, we choose *α* = 0.15 and *γ* = 0.8.

In each iteration, a data set $\left( {s_{t} ,R_{t} ,y_{t} } \right)$ is generated and stored in an experience pool, *D*. The objectives of the RL is to train the DNN to minimize the following loss function:

20 $$ L = E_{D} \left[ {(R_{t} + \gamma \mathop {\max }\limits_{{y_{t} }} Q\left( {s_{t} ,y_{t} ;\theta_{t}^{ - } } \right){ } - { }Q\left( {s_{t} ,y_{t} ;\theta_{t} } \right))^{2} } \right] $$

where $E\left[ {} \right]$ is the mathematical expectation, $\theta_{t}^{ - }$ is the parameter of the target DNN. This optimization can be solved using gradient descent method. It converges when $Q\left( {s_{t} ,y_{t} ;\theta_{t} } \right)$ reaches $\left( {R_{t} + \gamma \mathop {\max }\limits_{{y_{t} }} \left( {s_{t} ,y_{t} ;\theta_{t}^{ - } } \right)} \right)$.

The process of RL consist of the following steps:

Step 1: Initialize the action value function $y_{t}$, initialize the experience pool *D*, initialize the DNN parameter $\theta_{i}$, set the value of the action *Q* as a random number, and set $\theta_{i}^{ - } = \theta_{i}$;
Step 2: Enter the notes $y_{t}$ generated by LSTM (refer to Eq. (9)) as the initial value of $y_{t}$;.
Step 3: Compute the reward, $R_{t}$, using Eq. (15) and the average rate of return, $\overline{{R_{t} }}$, using Eq. (17);
Step 4: Compute the value of action, $Q\left( {s_{t} ,y_{t} ;\theta_{t} } \right),$ using Eq. (18) and the target Q-value, $\hat{Q}\left( {s_{t} ,y_{t} ;\theta_{t} } \right)$, using Eq. (19);
Step 5: Solve the loss function defined in Eq. (20) to get $\left( {s_{t} ,R_{t} ;{ }\theta_{t} } \right)$, and put it into the experience pool *D*;
Step 6: If the target is reached ($\overline{{R_{t} }} 1$ and *L* → 0), stop; else update ${ }\theta_{t}^{ - } = \theta_{t} ,t = t + 1$, goto Step 3.

## Summary of the training process

**Figure 9:**
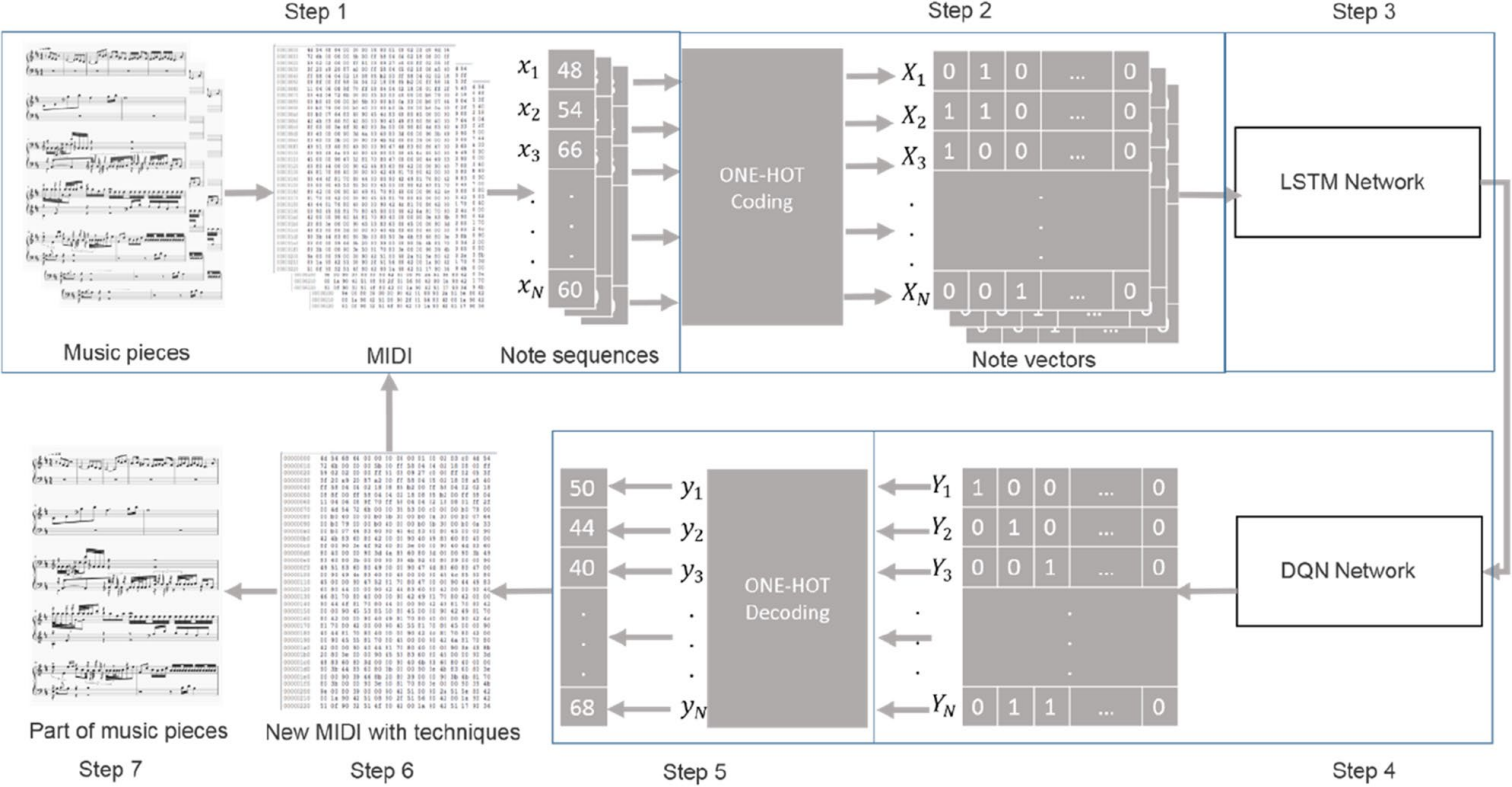
The training processes.

Figure 9 shows the training process of our new method. It consists of 7 steps:

Step 1: Covert the music notes to MIDI format, through which a piece of music can be represented as a matrix: **x** = {$x_{t,j}$, *t* = 1, 2,…, *N*, *j* = 1, 2,…, 128};
Step 2: Encode the data using One-Hot coding, which gives the input matrix: **X** = {$X_{t,j}$, *t* = 1, 2, …, *N*, *j* = 1, 2,…,128};
Step 3: Feed the data to the LSTM network described in Fig. 7;
Step 4: Feed the data to the DQN network described in Fig. 8, which gives the output matrix: **Y** = {$Y_{t,j}$, *t* = 1, 2, …,*M*, *j* = 1, 2,…,128};
Step 5: Decode the data, which gives **y** = {$y_{t,j}$, *t* = 1, 2, …, *M*, *j* = 1, 2, …, 128};
Step 6: Convert back to MIDI format, when necessary it can be fed into Step 1 for training again;
Step 7: Convert to the music note.

The computer program is written in Python using the following tools in the Python toolkit: TensorFlow V1.5, Keras V2.1.5, Music21 V5.2.0, Torch V1.8.0, and Mido V1.2.0.

## Post-processing and experiment results

### Converting the music with Guzheng timbre

As shown in the previous section, we use LSTM to generate Guzheng music and use RL to enhance the generated Guzheng music. However, the music is in piano timbre. Therefore, a post-processing step is necessary.

It is known that MIDI has many built-in timbre settings for different music instruments and sounds, but not for Guzheng. As discussed in Sect. 2, we generate the Guzheng music using piano timbre. To convert the piano timbre to Guzheng timbre we used a freeware “MIDI Cai Hong Gang Qi (MIDI彩虹钢琴)” for the conversion (https://www.cnblogs.com/qingjun1991/p/4971514.html).

**Figure 10:**
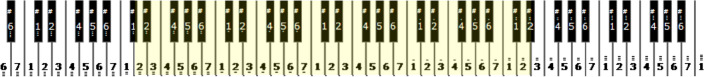
The correspondents between piano and Guzheng (in yellow).

It shall be noted that piano and Guzheng have different range of sound. Figure 10 shows the correspondents between the piano keyboard and Guzheng strings, marked in yellow. The 21 basic Guzheng sounds corresponds the 21 tones on the piano with the central C key of the piano corresponds to the 12th string of Guzheng. As we use the piano MIDI to generate the music, it is possible that some music notes are out of the range of the Guzheng. In this case, these music notes will be simply deleted.

### Experiment results

**Figure 11:**
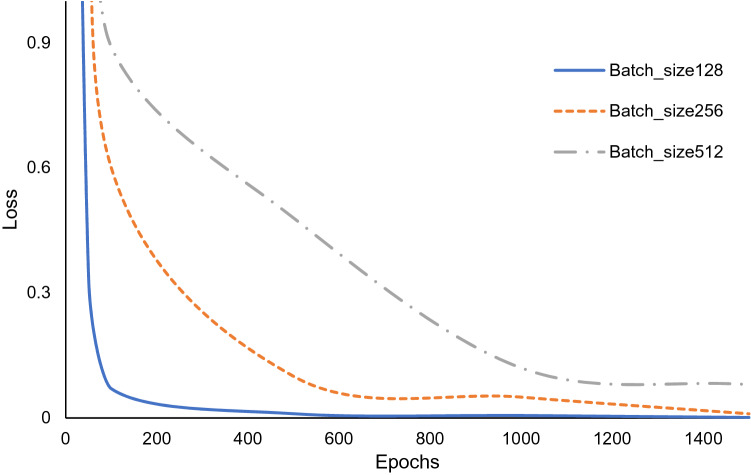
The relationship between the Loss and the Batch_size of LSTM.

Using the aforementioned method, new music pieces are generated. First, we decompose the MIDI files of the 31 Guzheng music into segments. Each segment has 1000 lines, covering 10 s. Then, we randomly pick these segments as the input of the LSTM. Upon receiving the input, the LSTM will generate an output, also lasts 10 s. The outputs are evaluated using the loss function defined in Eq. (10). The number of inputs is referred to as the Batch size. The larger the Batch size, the better the LSTM can learn, but the more the computation load. Figure 11 shows the loss function against the batch sizes of 128, 256 and 512. From the figure, it is seen that the larger Batch size require longer time to converge.

**Figure 12:**
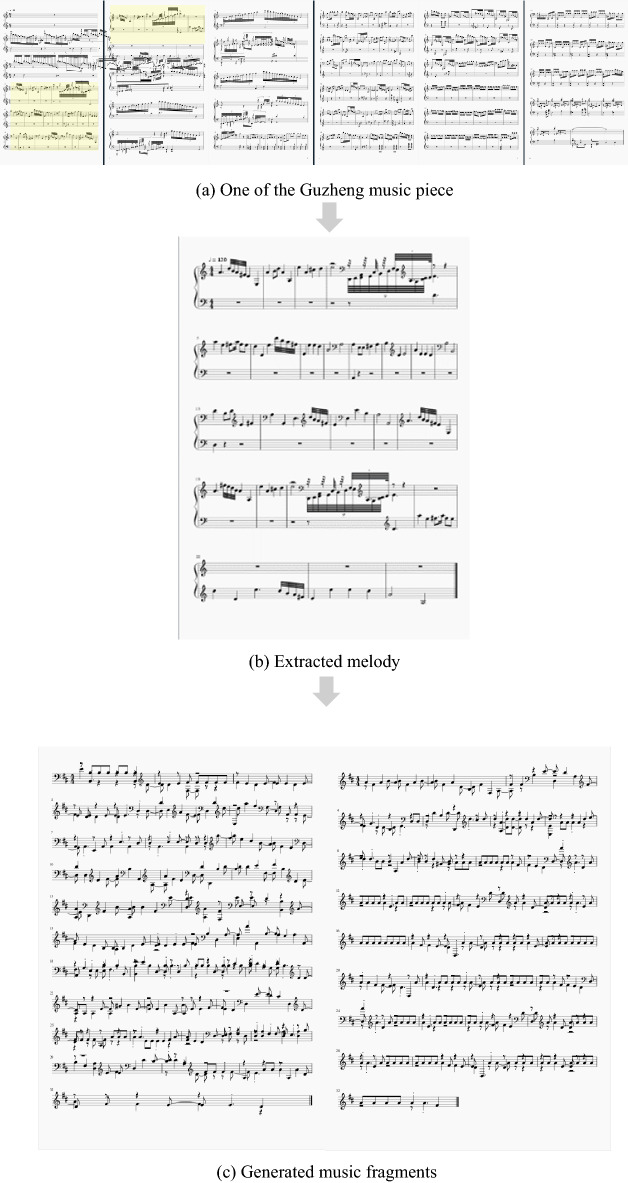
A sample music generated by LSTM with extracted melodies.

The generated music pieces are usually lack of melody and Guzheng characteristics. Thus, as discussed in “Extraction of the melodies” section, we extract the melodies from the training samples pieces and mixed them with the original 31 Guzheng music pieces as the training samples and run the LSTM again as illustrated in Fig. 12.

**Figure 13:**
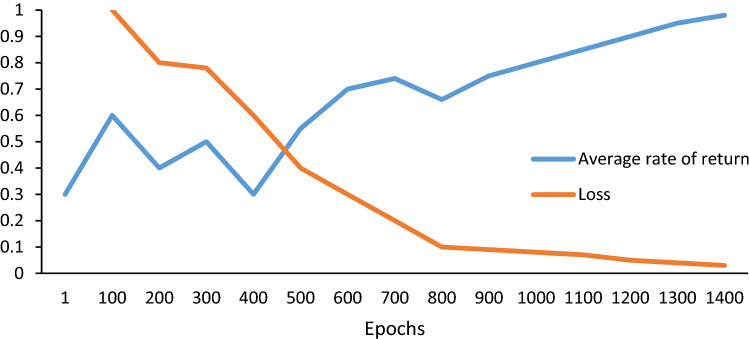
The relationship between the loss and the average rate of return of RL.

Then, we use RL to add the Guzheng characteristics. The convergence of the RL is evaluated using the average rate of return (the average ratio of the total revenue and the optimal revenue in the current training session) and the loss function defined by Eq. (20). As shown in Fig. 13, its coverages after 1400 epochs.

**Figure 14:**
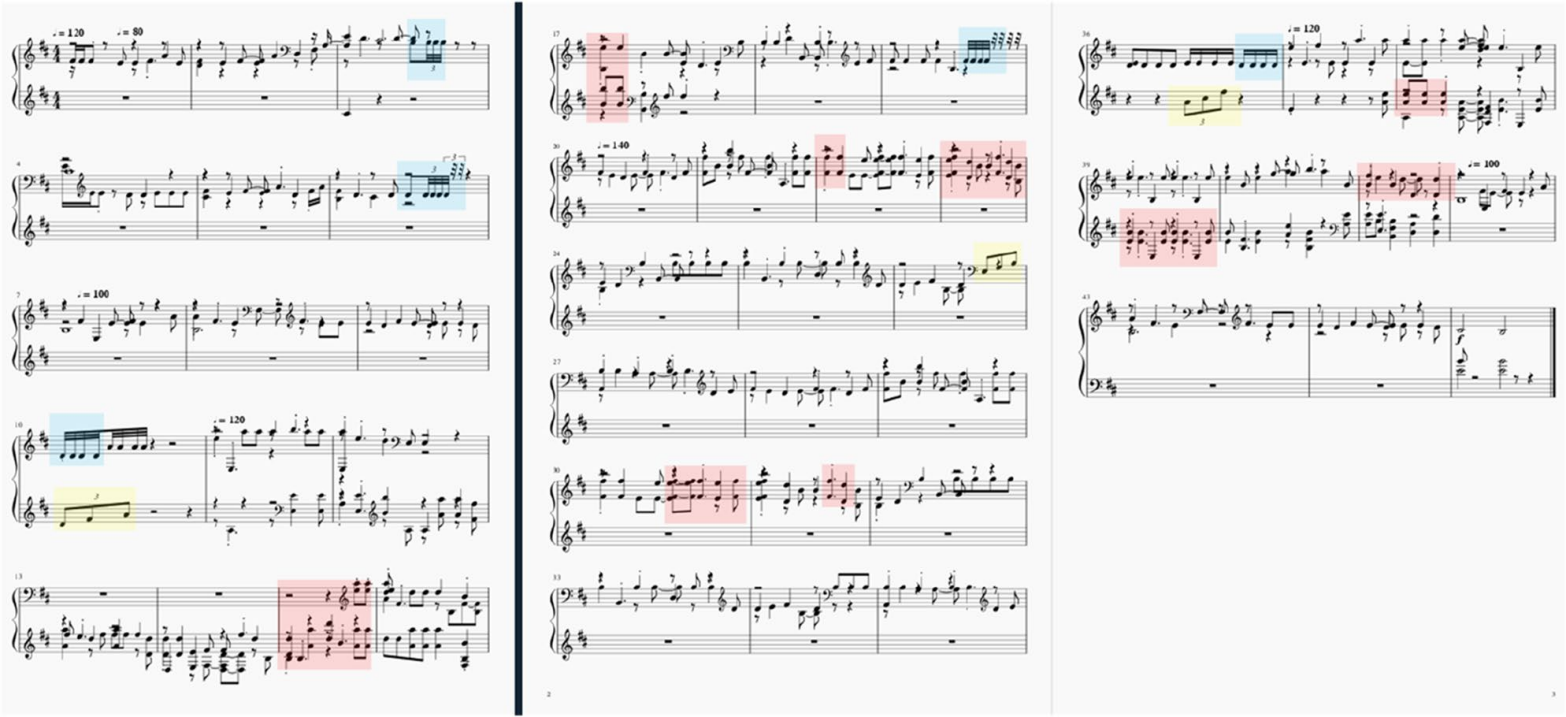
Generated music process.

Figure 14 shows one of the completed Guzheng music piece in which the Guzheng characteristics are marked: blue represents finger shake, yellow represents arpeggio and pink represents big & small pinch.

A number of music pieces have been generated. Three typical examples are upload on the webpage: https://www.bilibili.com/audio/am33421435?type=7. For comparison, a number of music pieces generated using LSTM alone and using GAN are also generated and uploaded on https://www.bilibili.com/audio/am33419632?type=7.

### Experiment result evaluation

It is known that the quality of a music piece is rather difficult to evaluate. Thus, we use both the mathematical method and the subjective method to evaluate.

The mathematical method is the note accuracy method^27^: given a music piece, deleting the last *N* notes, re-inputting into the trained model and regenerating the last *N* notes, then comparing the newly generated notes to the original notes. If the newly generated notes are similar to the original notes, then the trained model is considered good. Assuming that the generated notes are $\overline{y}_{t,j}$ and the original notes is $y_{t,j}$, the note accuracy, *A*, can be expressed as follows:

21 $$ A = \frac{{\mathop \sum \nolimits_{j = 1}^{M} \mathop \sum \nolimits_{t = 1}^{N} p\left( {y_{t,j} ,\overline{y}_{t,j} } \right)}}{MN} $$

where *j* = 1, 2,…, *M* denote test samples, *t* denotes the notes in the test sample, *N* is the number of notes and is set to 10, 20 and 25 respectively, and

$$ p\left( {y_{t,j} ,\overline{y}_{t,j} } \right) = \left\{ {\begin{array}{*{20}c} {1,} & {y_{t,j} = \overline{y}_{t,j} } \\ {0,} & {y_{t,j} \ne y_{t,j} } \\ \end{array} } \right\} $$

We used *M* = 5 generated music pieces to test our LSTM + RL model and the results are shown in Table 4. From the table, it is seen that our model has a good note accuracy.

**Table 4:** The note accuracy under different lengthen of nodes.

*N*	Accuracy (× 100%)
10	80%
20	75%
25	68%

Besides, we use the same method to test the effectiveness of RL. This time, instead of the last *N* notes, we search for the Guzheng playing techniques appeared in the music piece. Table 5 shows the experiment results on 10 generated music pieces. From the table, it is seen that using LSTM + RL gets much better results than that of using LSTM alone.

**Table 5:** The accuracy comparison between LSTM and LSTM + RL.

Characteristic	LSTM (× 100%)	LSTM + RL (× 100%)
Arpeggio	0.1%	6%
Finger shake	0.1%	8%
Big pinch & Small pinch	0.1%	17%
Repeating notes	11%	3%

**Figure 15:**
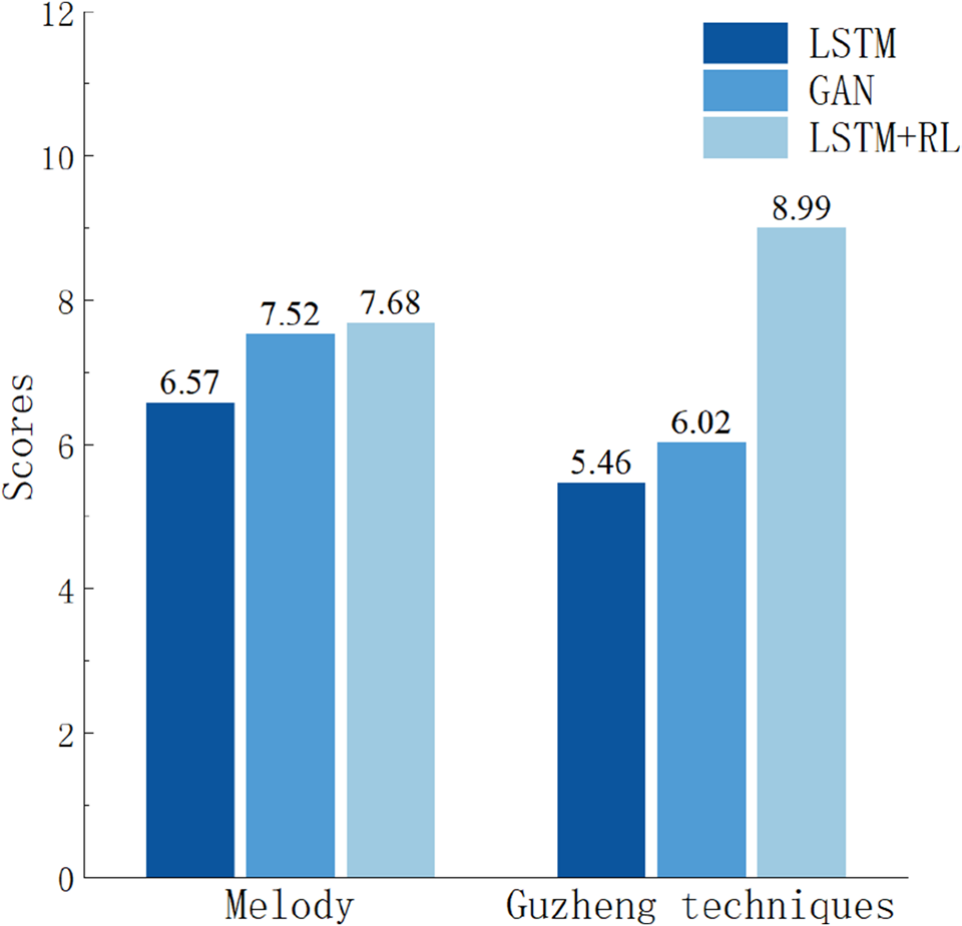
The subjective evaluation scores in melody and Guzheng playing techniques.

The subjective evaluation is based on the interview of 10 experienced Guzheng players^28,29^. We prepared a set of Guzheng music pieces, including three pieces generated by LSTM, three pieces generated by LSTM + RL model and three pieces generated by GAN. The interviewees are asked to rank both the melody and the Guzheng playing techniques on the scale of 10. The evaluation results are summarized in Fig. 15. As shown in the figure, compared to LSTM and GAN, the LSTM + RL has the best performance in melody. This implies that the music generated by LSTM + RL is better in resembling the traditional Guzheng music. Also, LSTM + RL generates much more Guzheng playing techniques. Consequently, it is said that our Guzheng music is indeed close to the real Guzheng music.

## Conclusions and future work

This paper presents a new method for composing Guzheng music using LSTM and RL. Based on the discussions above, following conclusions can be drawn:

1. The presented LSTM + RL method is effective. According to the evaluation by note accuracy method and by 10 skilled Guzheng players, the generated music pieces is closely resembling the classic Guzheng music.
2. The key to the success is to extract the melody from the training samples using LSTM as well as adding Guzheng characteristics using RL. This LSTM + RL combination makes the generated music more like a Guzheng music instead of mere imitation.
3. The presented method is efficient. Using the MIDI format, the training and music generation take only a few hours in a PC computer.

We expect that the same method can be applied to compose music for other traditional Chinese music instruments, such as Pipa (琵琶), Guqin (古琴), Lusheng (芦笙) and etc.

For the future work, two issues can be pursued.

1. In the training, the sampled music piece is divided into multiple segments, each has 10 s in length. While this is simple and effective, it may miss the long-term correlation in a music piece. The use of longer segments (e.g., 30 s or longer) may be beneficial for slow mode music, though, the training will be longer.
2. The emotional features of the music pieces can be extracted, classified and labeled in the training process to further optimize the generated music.

## Data Availability

The datasets used and/or analysed during the current study available from the corresponding author on reasonable request.
